# Bacterial vaginosis in early pregnancy and birth outcomes: Evidence from a prospective cohort in the middle belt of Ghana

**DOI:** 10.1371/journal.pone.0354974

**Published:** 2026-07-31

**Authors:** Dennis Gyasi Konadu, Alex Kwame Owusu-Ofori, David Kwame Dosoo, Zuwera Yidana, Gladys Matuamo Ngo, Philomina Nkansah Nfum, Richard Joshua Tetteh, Dennis Adu-Gyasi, Kwaku Poku-Asante

**Affiliations:** 1 Kintampo Health Research Centre, Kintampo, Bono East, Ghana; 2 Department of Clinical Microbiology, School of Medical Science, Kwame Nkrumah University of Science and Technology, Kumasi, Ghana; Universidade dos Açores Departamento de Biologia: Universidade dos Acores Departamento de Biologia, PORTUGAL

## Abstract

**Background:**

Bacterial vaginosis (BV) is highly prevalent in women of African origin and has been associated with adverse birth outcomes (ABO), particularly preterm birth (PTB). However, evidence from sub-Saharan Africa remains limited, and while a previous study conducted in this area examined the prevalence of BV, its impact on pregnancy outcomes was not assessed. This study assessed the prevalence and risk factors of BV in early pregnancy and examined its association with ABO in the middle belt of Ghana.

**Methods:**

This analysis was nested within a prospective cohort of pregnant women in the Kintampo North Municipality and Kintampo South District. Pregnant women <20 weeks’ gestational age were screened, consented, enrolled and followed through delivery until one year after birth. At enrolment, sociodemographic, behavioural, and obstetric data were collected, along with biological samples, including vaginal swabs for Gram staining and Nugent scoring. BV status was categorized as BV-negative (0–6) or BV-positive (7–10) and ABO included PTB (<37 weeks), low birthweight (LBW < 2.5 kg), and foetal loss. Data were double-entered in REDCap and analysed in R. Associations between BV and ABO were assessed using chi-square tests, and logistic regression was used to identify risk factors for BV.

**Results:**

Of 1,180 pregnant women enrolled, 355 (30.1%) were BV-positive. PTB (9.9% vs. 8.7%; p = 0.50), LBW (13.8% vs. 12.0%; p = 0.39), and foetal loss (4.5% vs. 2.8%; p = 0.14) were all higher among BV-positive women, but none reached statistical significance. Younger age and unmarried status were associated with BV. Unmarried women (AOR 1.63; 95% CI: 1.20–2.22) and those reporting one sexual encounter per week (AOR 1.55; 95% CI: 1.01–2.37) had increased odds of BV.

**Conclusions:**

BV is highly prevalent in early pregnancy in this Ghanaian cohort, particularly among younger and unmarried women, but was not significantly associated with ABO. Targeted screening among high-risk groups is necessary, and future molecular approaches may better characterise BV subtypes linked to ABO in African settings.

## Introduction

Bacterial vaginosis (BV), an endogenous infection, is a dysbiosis of the vaginal microbiota characterized by a decrease in *Lactobacillus spp*. and an overgrowth of anaerobic bacteria such as *Gardnerella spp, Prevotella spp, and Mobiluncus spp*., [1]. This dysbiosis has significant implications in pregnancy given its association with adverse maternal and foetal outcomes [2,3]. In sub-Saharan Africa, the burden of BV among pregnant women remains substantial [4–6], coinciding with elevated rates of preterm birth (PTB), low birth weight (LBW), and perinatal morbidity and mortality [7].

BV is prevalent in women of African origin with rates such as 26.3% [5] in Democratic Republic of Congo, 30.9% in Ghana [6] and as high as 48.5% in Uganda [8]. A systematic review by Park et al (2024), reported that women of sub-Saharan Africa had the highest pooled prevalence of 25.1%, followed by 22.6% in Latin America and the Caribbean [4]. These rates indicate a high burden of BV in people of African origin and the need to develop measures to mitigate its effect in pregnancy.

Although several methods exist for BV diagnosis, including clinical Amsel’s criteria and molecular approaches, the Nugent scoring method is considered the gold standard in research settings due to its objectivity and reproducibility compared to clinical methods [9,10].

Preterm birth (PTB) defined as birth before 37 completed weeks of gestation, is the leading cause of neonatal morbidity and mortality globally. Its aetiology is multifactorial, with established risk factors including prior preterm birth, maternal infection, inadequate antenatal care, extremes of maternal age, and poor nutritional status [11]. Studies has shown pre-pregnancy underweight (BMI < 18.5 kg/m²) and maternal anaemia (haemoglobin <11 g/dL) independently increase the risk of preterm birth [11]. Globally, about 13.4 million (9.8%) babies are born preterm with an estimated 900,000 deaths due to preterm complications [7]. PTB rate is highest in Southern Asia (13.2%), followed by Sub-Saharan Africa (10.1%) [12]. In Ghana, the PTB rate varies from as low as 4.7% [13] in Cape Coast Teaching Hospital to as high as 18.9% [14] at the Korle Bu Teaching Hospital. Beyond preterm birth, BV has been linked to other adverse outcomes including preterm premature rupture of membranes (PPROM), low birth weight (LBW), intrauterine infection, and chorioamnionitis.

Genital tract infection, particularly bacterial vaginosis (BV), occupies a central position among modifiable infectious risk factors. BV-associated anaerobic bacteria may ascend from the lower genital tract to the foetal membranes, leading to subclinical inflammation (chorioamnionitis), weakening of membranes, and induction of early labour. These mechanisms may underlie BV’s role in preterm labour, PPROM, and intrauterine infection [15].

Several meta-analyses and systematic reviews have reported associations between BV and adverse outcomes including preterm birth (PTB) in pregnant women diagnosed with BV, though the evidence base remains heterogeneous, and high-quality studies are still needed to establish a consistent causal relationship in diverse populations. [15–18].

Considering the high prevalence of BV documented in this area (30.9%) [6] and the reported associations between BV and adverse pregnancy outcomes in several studies, there is a compelling need to examine its impact on pregnancy outcomes locally. While few studies from sub-Saharan Africa have examined this association, findings remain inconsistent across settings [2,5]. Evidence from Ghana specifically remains largely absent, with existing local studies limited to prevalence estimation. Building on a previous prevalence study conducted in this area, this study therefore investigated the association between BV, diagnosed using the Nugent score-defined BV, and adverse birth outcomes among pregnant women in the middle belt of Ghana.

## Materials and methods

### Ethical approval

This study was nested into the PRISMA MNH study (Pregnancy Risk Stratification Innovation and Measurement Alliance – Maternal Neonatal Health) carried out in Kintampo North Municipality and Kintampo South District, Bono East Region, Ghana [19]. Ethical approval and oversight were obtained from Ghana Health Service Ethics Review Committee (GHSERC:019/09/20) and the Kintampo Health Research Centre Institutional Ethics Committee (KHRCIEC/2020-17). Written informed consent, assent and parental consent were sought from all study participants as appropriate prior to enrolment. All experiments were performed in accordance with the Declaration of Helsinki. Enrolment of study participants was initiated between 28 December 2022 and 18 December 2025.

### Study design, population and area

The PRISMA MNH study was a large prospective cohort of pregnant women conducted in the Kintampo North Municipality and Kintampo South District, located in the forest–savannah transitional belt of central Ghana. The two districts have a combined resident population of approximately 156,145 and are predominantly rural, characterized by dispersed settlements and limited access to advanced healthcare services. The population is largely youthful, with a substantial proportion of women of reproductive age who utilize antenatal care services. However, routine laboratory diagnosis of reproductive tract infections, including bacterial vaginosis (BV), remains limited, with most cases managed syndromically. Key risk factors include rural residence, socio-economic constraints, and gaps in diagnostic capacity, all of which contribute to delayed detection and treatment and, consequently, an increased risk of adverse pregnancy outcomes.

### Recruitment of participants

A total of 1,180 pregnant women residing in the Kintampo North Municipality and Kintampo South District were recruited through an active community-based surveillance system. Trained fieldworkers stationed within communities conducted monthly household visits to identify new pregnancies among women of reproductive age. Women identified as potentially pregnant were informed about the study objectives, procedures, risks, and benefits in the local language. Those who expressed interest underwent assessment by a study midwife, who confirmed pregnancy viability and estimated gestational age using ultrasound scan prior to enrolment.

### Eligibility criteria

Women were eligible for enrolment if they met all of the following inclusion criteria: (i) confirmed viable intrauterine pregnancy on ultrasound at the time of screening; (ii) gestational age less than 20 weeks; (iii) aged between 18 and 49 years; (iv) resident within the Kintampo North Municipality or Kintampo South District; and (v) willing and able to provide written informed consent and available for follow-up through delivery and up to one year postpartum.

Women were excluded if they met any of the following criteria: (i) gestational age 20 weeks or greater at screening; (ii) non-viable or ectopic pregnancy confirmed on ultrasound; (iii) unable or unwilling to provide informed consent; or (iv) not resident within the study area or unlikely to remain available for follow-up through delivery.

### Study procedures

After the participants were enrolled, a study questionnaire was administered to gather data on their demographics, symptoms of vaginal infection and vaginal hygiene practices. Study midwives performed physical examination including anthropometric measurements such as weight and height for body mass index (BMI) and mid-upper arm circumference (MUAC). Three vaginal swabs, urine, and blood samples were collected at enrolment and transported in conditioned transport boxes with ice packs to the Seth Owusu-Agyei Medical Laboratory at the Kintampo Health Research Centre for analysis or storage, as appropriate. At delivery, obstetrical parameters such as type of delivery, and neonatal examination for anthropometric measurements (length, weight and head circumference) were taken and recorded by qualified midwives.

### Study specific laboratory tests

Upon receipt of vaginal swabs in the laboratory, Nugent criteria and wet mount were performed. Briefly, one swab was used to make a smear on a glass slide, heat fixed and Gram stained for microscopy. The Gram-stained slides were read by two independent readers blinded to each other’s results. Concordance was defined as agreement between both readers on the Nugent score category either BV-negative (score 0–3), intermediate (4–6) or BV-positive (score 7–10). If there were discordant readings, the two slide readers reviewed the slide together and arrived at a consensus. For the Nugent scoring system, the average of ten high-power fields is scored for large Gram-positive rods, Gram variable rods and Gram-negative curved rods. A Gram stain score of 0–3 was considered “normal”, a score of 4–6 was considered “intermediate” and a score of 7–10 was considered positive for BV. Participants with an intermediate score were also categorized as normal for the analysis. Presence of pus cells, clue cells and yeast cells and/or pseudohyphae were also documented. BV-positive participants were not treated as part of the study protocol. However, women diagnosed with BV were referred for routine antenatal care, where treatment was provided at the discretion of the attending clinicians.

Another swab was used for the wet mount by adding drops of saline to the swab in a glass tube. A drop of the saline with the vaginal fluid was placed on a glass slide and observed under the microscope at 10X and 40X objective lenses for epithelial cells, pus cells, red blood cells, *Trichomonas vaginalis*, yeast cells and clue cells.

### Data management and analysis

All questionnaire and laboratory data were double-entered into REDCap version 16.0.8 (Research Electronic Data Capture) hosted at KHRC, a secure web-based application designed to support data capture for research studies [20,21]. Any discrepancies between the two datasets were resolved by referencing the original source documents, after which a final cleaned dataset was generated. Statistical analysis was conducted using R version 4.5.2.

Participants characteristics were summarized using descriptive statistics. Categorical variables were presented as frequencies and proportions, while continuous variables were summarized using medians and interquartile ranges. For the BV status analysis, Nugent scores 0–3 (normal microbiota) and 4–6 (intermediate microbiota) were combined and classified as “BV-negative (0-6),” while Nugent scores 7–10 were classified as BV-positive. This binary categorization was adopted in line with clinical diagnostic cutpoint used in Nugent-based epidemiological studies of BV and pregnancy outcomes, to improve statistical power and facilitate interpretation of clinically relevant outcomes. Given that vaginal microbiota was assessed at a single time point, the transitional nature of intermediate flora could not be reliably distinguished.

To examine the relationship between BV and adverse birth outcomes such as preterm birth, low birthweight, and foetal loss. BV status was treated as the primary independent variable, while each birth outcome served as a dependent variable. Associations between BV and these outcomes were assessed using chi-square tests.

Risk factors for BV were assessed using a series of logistic regression models. Rather than relying solely on automated model selection, we utilized a forced-entry method in which all pre-specified variables were retained in the model regardless of statistical significance. Factors such as sociodemographic background, behavioural patterns, and obstetric history were included in the multivariable analysis due to their documented clinical importance. By maintaining a consistent modelling approach, we provided a comparative view of crude and adjusted associations for all primary variables of interest.

## Results

### Sociodemographic and clinical characteristics of participants

A total of 1,180 pregnant women were enrolled in the study. Based on Nugent scoring, 825 (69.9%) were classified as BV-negative, while 355 (30.1%) had BV-positive microbiota. The distribution of sociodemographic, behavioural, clinical and obstetric characteristics across vaginal microbiota categories is presented in Table 1.

**Table 1:** Sociodemographic, obstetric, behavioural, and clinical characteristics of the study population stratified by Bacterial Vaginosis Status.

	Overall N = 1,180 (100%)	BV Negative n = 825 (69.9%)	BV Positive n = 355 (30.1%)
***Sociodemographic Characteristics***			
**Age Group**			
≤20	152 (13%)	83 (10%)	69 (19%)
21-29	580 (49%)	405 (49%)	175 (49%)
30+	448 (38%)	337 (41%)	111 (31%)
**Marital Status**			
Married	809 (69%)	602 (73%)	207 (58%)
Not Married	371 (31%)	223 (27%)	148 (42%)
**Ethnic Group**			
Akan/Bono	145 (12%)	95 (12%)	50 (14%)
Mo	97 (8.2%)	68 (8.2%)	29 (8.2%)
Northern Tribes	929 (79%)	657 (80%)	272 (77%)
Others	9 (0.8%)	5 (0.6%)	4 (1.1%)
**Religion**			
Christian	727 (62%)	505 (61%)	222 (63%)
Muslim	418 (35%)	293 (36%)	125 (35%)
Other	35 (3.0%)	27 (3.3%)	8 (2.3%)
**Employment Status**			
Employed	817 (69%)	588 (71%)	229 (65%)
Unemployed	363 (31%)	237 (29%)	126 (35%)
**Wealth Quintile**			
Least Poor	226 (19%)	163 (20%)	63 (18%)
Less Poor	249 (21%)	191 (23%)	58 (16%)
Poor	221 (19%)	147 (18%)	74 (21%)
More Poor	234 (20%)	155 (19%)	79 (22%)
Most Poor	250 (21%)	169 (20%)	81 (23%)
***Obstetric Characteristics***			
**Parity**			
High multiparity	75 (6.4%)	59 (7.2%)	16 (4.5%)
Low multiparity	747 (63%)	526 (64%)	221 (62%)
Nulliparity	358 (30%)	240 (29%)	118 (33%)
**Previous Miscarriage**			
No	1,035 (88%)	721 (87%)	314 (88%)
Yes	145 (12%)	104 (13%)	41 (12%)
**BMI Categorization**			
Normal	761 (64%)	526 (64%)	235 (66%)
Obese	93 (7.9%)	66 (8.0%)	27 (7.6%)
Overweight	267 (23%)	193 (23%)	74 (21%)
Underweight	59 (5.0%)	40 (4.8%)	19 (5.4%)
***Behavioural Characteristics***			
**Vaginal Douching**			
No	1,114 (94%)	775 (94%)	339 (95%)
Yes	66 (5.6%)	50 (6.1%)	16 (4.5%)
**Smoking**			
No	1,177 (99.7%)	822 (99.6%)	355 (100%)
Yes	3 (0.3%)	3 (0.4%)	0 (0%)
**Sex Intercourse Frequency per Week**			
>2	182 (15%)	140 (17%)	42 (12%)
2	417 (35%)	292 (35%)	125 (35%)
1	334 (28%)	221 (27%)	113 (32%)
0	247 (21%)	172 (21%)	75 (21%)
**New Sexual Partner in Past 3 months**			
No	1,155 (98%)	810 (98%)	345 (97%)
Yes	25 (2.1%)	15 (1.8%)	10 (2.8%)
**Antimicrobial Use in the past 3 weeks**			
No	1,023 (87%)	722 (88%)	301 (85%)
Yes	157 (13%)	103 (12%)	54 (15%)
***Clinical and Microbiological Findings***			
**Haemoglobin Categorization**			
Normal	422 (36%)	303 (37%)	119 (34%)
Mild	534 (45%)	364 (44%)	170 (48%)
Moderate	215 (18%)	152 (18%)	63 (18%)
Severe	9 (0.8%)	6 (0.7%)	3 (0.8%)
**HIV Status**			
Negative	1,175 (99.6%)	822 (99.6%)	353 (99.4%)
Positive	5 (0.4%)	3 (0.4%)	2 (0.6%)
**Presence of Clue Cells**			
No	949 (80.4%)	789 (95.6%)	160 (45.1%)
Yes	231 (19.6%)	36 (4.4%)	195 (54.9%)
**Presence of Pus Cells**			
≤10 cells/HPF	1,012 (85.8%)	732 (88.7%)	280 (78.9%)
>10 cells/HPF	168 (14.2%)	93 (11.3%)	75 (21.1%)
**Presence of Candida cells**			
No	842 (71.4%)	602 (73.0%)	240 (67.6%)
Yes	338 (28.6%)	223 (27.0%)	115 (32.4%)
**Presence of *T. vaginalis***			
No	1,173 (99.4%)	820 (99.4%)	353 (99.4%)
Yes	7 (0.6%)	5 (0.6%)	2 (0.6%)
**Any Symptom of Vaginal Infection**			
No	831 (70.4%)	595 (72.1%)	236 (66.5%)
Yes	349 (29.6%)	230 (27.9%)	119 (33.5%)

n(%) = frequency (percent).

**Fig 1:**
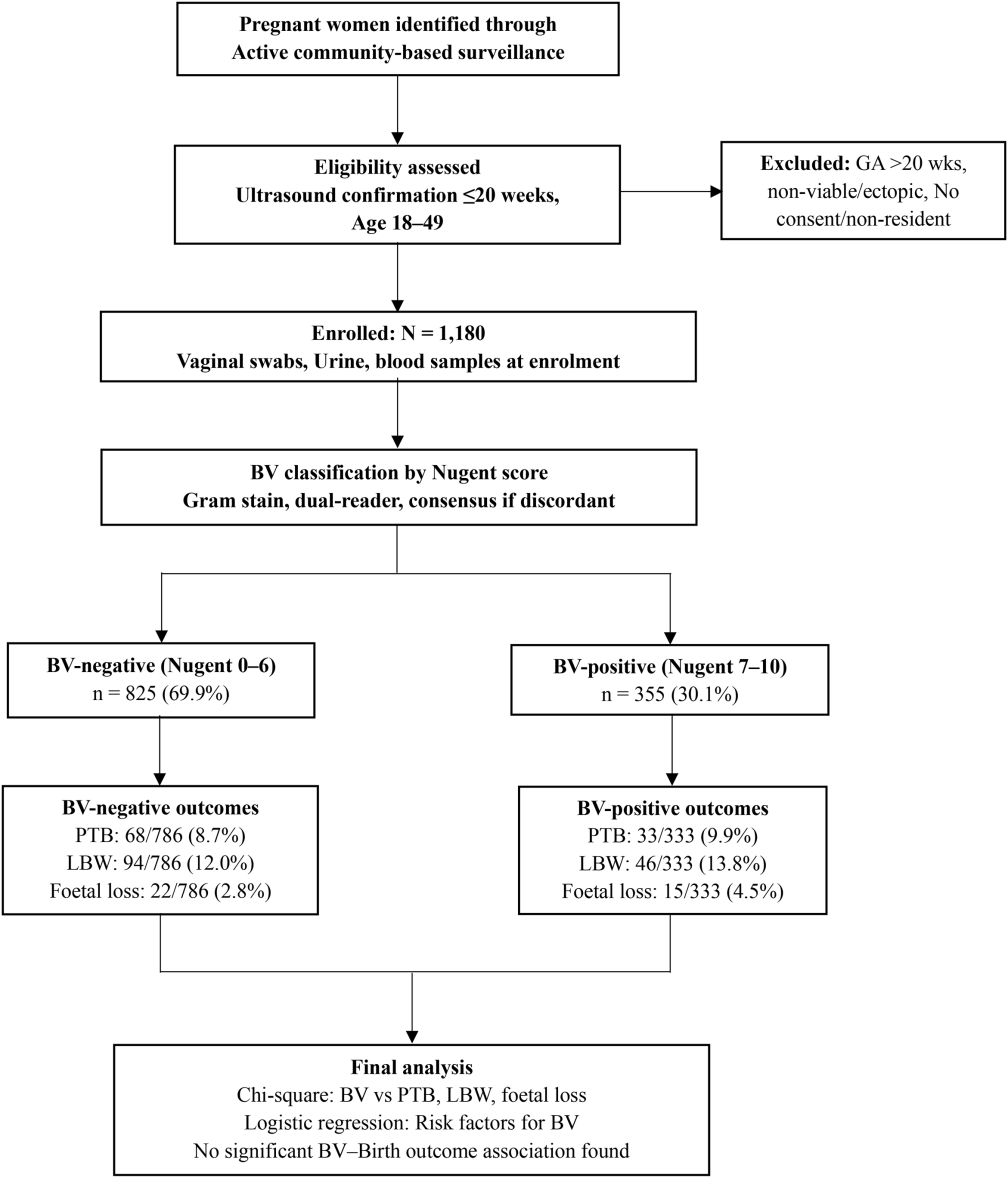
Flowchart of study. GA = Gestational age BV = Bacterial Vaginosis, PTB = Preterm Birth, LBW = Low Birth Weight.

Age distribution differed between groups, with a higher proportion of BV-positive women aged ≤20 years (19%) compared to BV-negative women (10%). Conversely, women aged ≥30 years were more represented in the BV-negative group (41%) than in the BV-positive group (31%). Marital status showed a notable difference, with 42% of BV-positive women being unmarried compared to 27% among BV-negative participants. Employment status, BMI categories, parity, douching, and HIV status showed similar distributions across groups. Recent antimicrobial use was slightly higher among BV-positive women (15%) compared to BV-negative women (12%). Most participants reported no new sexual partner in the preceding three months (98% in BV-negative vs. 97% in BV-positive). Clue cells were significantly more common among BV-positive women (54.9%) than BV-negative women (4.4%). Co-infection with *Candida* spp. And *T. vaginalis* was observed in 28.6% and 0.6% of participants respectively. Overall, 349 women (29.6%) reported at least one symptom of vaginal infection (abnormal discharge, itching, abdominal pain, or painful urination). The prevalence of symptoms was higher among BV-positive women than BV-negative women (33.5% vs. 27.9%); however, most participants in both groups were asymptomatic (Fig 1).

### Vaginal microbiota and birth outcomes

Pregnancy outcomes stratified by Nugent score are summarized in Table 2. Of the 1,180 women enrolled, birth outcome data were available for 1,119 (94.8%), the remaining 61 women had left the study, delivered outside the catchment area or facility, or experienced a miscarriage. The overall prevalence of preterm birth (<37 weeks) was 9.0%. Preterm delivery occurred in 9.9% of BV positive women and 8.7% of BV negative women, with no significant difference between the groups (p = 0.500). Low birthweight (<2.5 kg) affected 12.5% of all births and occurred at similar frequencies in both BV-negative (12.0%) and BV-positive (13.8%) women (p = 0.390). Foetal loss occurred in 3.3% of pregnancies overall, with a slightly higher proportion in the BV positive group (4.5%) compared to the BV negative group (2.8%), although this difference was not statistically significant (p = 0.140). All the adverse birth outcomes, Preterm birth (9.9% vs. 8.7%; p = 0.50), low birthweight (13.8% vs. 12.0%; p = 0.39), and foetal loss (4.5% vs. 2.8%; p = 0.14) were all higher among BV-positive women, but none reached statistical significance.

**Table 2:** Pregnancy outcomes stratified by Bacterial Vaginosis Status.

	Overall N = 1,119	BV Negative n = 786	BV Positive n = 333	p-values
**Gestational Age Categorization**				0.500
Preterm (<37)	101 (9.0%)	68 (8.7%)	33 (9.9%)	
Term (≥37)	1,018 (91%)	718 (91.3%)	300 (90.1%)	
**Birth Weight**				0.390
Low birthweight (LBW)	140 (12.5%)	94 (12.0%)	46 (13.8%)	
Normal Birthweight	979 (87.5%)	692 (88.0%)	287 (86.2%)	
**Birth Outcome**				0.140
Foetal loss	37 (3.3%)	22 (2.8%)	15 (4.5%)	
Live birth	1,082 (97%)	764 (97%)	318 (95%)	

Statistical comparison was performed using chi-square test. n (%) = counts (percent).

Birth outcome data were available for 1,119 of the 1,180 enrolled participants. Sixty-one women (5.2%) were excluded from the birth outcome analysis due to loss to follow-up, delivery outside the study area/facility, or miscarriage.

### Risk factors of bacterial vaginosis

The logistic regression analysis examining risk factors of BV is summarized in Table 3.

**Table 3:** Logistic regression analysis (Risk factors for BV).

Characteristic	Descriptive		Unadjusted			Adjusted		
	BV Negative N = 825	BV Positive N = 355	COR	95% CI	P-value	AOR	95% CI	P-value
**Age Category**								
≤20	83 (10%)	69 (19%)	1	—	—	1	—	—
21-29	405 (49%)	175 (49%)	0.52	0.36, 0.75	<0.001	0.59	0.39, 0.90	0.014
30+	337 (41%)	111 (31%)	0.40	0.27, 0.58	<0.001	0.53	0.33, 0.85	0.009
**Marital Status**								
Married	602 (73%)	207 (58%)	1	—	—	1	—	—
Not Married	223 (27%)	148 (42%)	1.93	1.49, 2.51	<0.001	1.66	1.23, 2.25	0.001
**Religion**								
Christian	505 (61%)	222 (63%)	1	—	—	1	—	—
Muslim	293 (36%)	125 (35%)	0.97	0.75, 1.26	0.8	1.11	0.84, 1.49	0.4620
Other	27 (3.3%)	8 (2.3%)	0.67	0.28, 1.44	0.3	0.65	0.28, 1.52	0.3209
**Employment**								
Employed	588 (71%)	229 (65%)	1	—		1	—	
Unemployed	237 (29%)	126 (35%)	1.37	1.05, 1.78	0.021	1.07	0.80, 1.42	0.667
**Wealth Quintile**								
Least Poor	163 (20%)	63 (18%)	1	—	—	1	—	—
Less Poor	191 (23%)	58 (16%)	0.79	0.52, 1.19	0.3	0.78	0.51, 1.20	0.257
Poor	147 (18%)	74 (21%)	1.30	0.87, 1.95	0.2	1.25	0.82, 1.90	0.299
More Poor	155 (19%)	79 (22%)	1.32	0.89, 1.97	0.2	1.20	0.80, 1.82	0.376
Most Poor	169 (20%)	81 (23%)	1.24	0.84, 1.84	0.3	1.11	0.73, 1.67	0.627
**Parity**								
High multiparity	59 (7.2%)	16 (4.5%)	1	—	—	1	—	—
Low multiparity	526 (64%)	221 (62%)	1.55	0.89, 2.84	0.14	1.28	0.71, 2.35	0.420
Nulliparity	240 (29%)	118 (33%)	1.81	1.02, 3.38	0.050	1.12	0.58, 2.14	0.580
**BMI Category**								
Normal	526 (64%)	235 (66%)	1	—	—	1	—	—
Obese	66 (8.0%)	27 (7.6%)	0.92	0.56, 1.45	0.7	1.05	0.64, 1.71	0.861
Overweight	193 (23%)	74 (21%)	0.86	0.63, 1.17	0.3	0.93	0.67, 1.28	0.643
Underweight	40 (4.8%)	19 (5.4%)	1.06	0.59, 1.85	0.8	0.96	0.53, 1.72	0.889
**Vaginal Douching**								
No	775 (94%)	339 (95%)	1	—	—	1	—	—
Yes	50 (6.1%)	16 (4.5%)	0.73	0.40, 1.27	0.3	0.85	0.47, 1.57	0.623
**Sex Intercourse Frequency Per Week**								
>2	140 (17%)	42 (12%)	1	—	—	1	—	—
2	292 (35%)	125 (35%)	1.43	0.96, 2.15	0.084	1.20	0.85, 1.95	0.231
1	221 (27%)	113 (32%)	1.70	1.13, 2.59	0.011	1.55	1.01, 2.37	0.044
0	172 (21%)	75 (21%)	1.45	0.94, 2.27	0.095	0.97	0.54, 1.76	0.922
**New Partner Sexual in Past 3 months**								
No	810 (98%)	345 (97%)	1	—	—	1	—	—
Yes	15 (1.8%)	10 (2.8%)	1.63	0.67, 3.48	0.3	1.61	0.70, 3.80	0.255
**Antimicrobial Use in the past 3 weeks**								
No	722 (88%)	301 (85%)	1	—	—	1	—	—
Yes	103 (12%)	54 (15%)	1.26	0.88, 1.79	0.2	1.62	0.90, 2.88	0.105

Abbreviations: CI = Confidence Interval, OR = Odds Ratio.

### Sociodemographic predictors

Age was a significant risk factor of BV. Compared with adolescents aged ≤20 years, women aged 21–29 years had lower odds of BV (AOR = 0.59, 95% CI: 0.39–0.90, p = 0.014), and those aged ≥30 years also showed reduced odds (AOR = 0.53, 95% CI: 0.33–0.85, p = 0.009). Marital status remained significant after adjustment; unmarried women had a higher likelihood of BV (AOR = 1.66, 95% CI: 1.23–2.25, p = 0.001).

### Behavioural and reproductive characteristics

Sexual Intercourse frequency was associated with BV. Compared with women reporting more than two sexual encounters per week, those reporting one weekly encounter had increased odds of BV (AOR = 1.55, 95% CI: 1.01–2.37, p = 0.044). Other sexual frequency categories were not statistically significant. Vaginal douching, new sexual partners, and antimicrobial use were not associated with BV after adjustment.

### Other factors

BMI, parity, employment status, religion, and wealth quintile were not significantly associated with BV in either the unadjusted or adjusted models.

## Discussion

The prevalence of BV in this cohort (30.1%) is consistent with earlier work of 30.9% in Kintampo, Ghana [6] which shows no change in prevalence after a decade. It aligns with recent estimates pooled prevalence of 36.6% [22] and 25.1% [4], across sub-Saharan Africa, where prevalence remains among the highest globally. This high prevalence also reflects significant endemicity consistent with reports from other sub-Saharan African settings [5] and remains a pervasive reproductive health issue in the region [23].

Younger maternal age (Adolescents, ≤ 20 years) and unmarried women had markedly higher odds of BV, a finding echoed in recent African and global data showing that adolescents experience increased microbial instability due to hormonal changes, sexual exposure patterns, reduced access to reproductive health services and socioeconomic factors [24,25]. There is the need for targeted public health interventions addressing these vulnerable groups.

Marital status demonstrated a strong association with BV in our study. This association may reflect differences in sexual partnership patterns, with unmarried women potentially having multiple or new sexual partners, factors known to disrupt vaginal microbiota [26]. In African settings, where marriage often correlates with more stable sexual partnerships and potentially different reproductive health behaviours. However, this finding should be interpreted cautiously, as marital status may serve as a proxy for other unmeasured socioeconomic or behavioural factors.

Sexual frequency also showed a significant association with BV. This finding is somewhat paradoxical, as frequent sexual intercourse has been traditionally associated with increased BV risk due to repeated disruption of the vaginal environment and exposure to semen, which has an alkaline pH [27]. The relationship between sexual activity and BV is complex and may be influenced by partner-related factors, contraceptive use, and hygiene practices.

Notably, we found no significant associations between BV and several factors previously reported in other settings, including BMI, parity, douching practices, smoking, or wealth quintile. The lack of association with douching is particularly noteworthy, as douching has been consistently identified as a risk factor for BV in Western populations [27]. The very low prevalence of douching in our sample (5.6%) may have limited our ability to detect an association, or alternatively, may reflect cultural differences in vaginal hygiene practices that do not substantially impact BV risk in this population.

Although BV-positive women were more likely to report symptoms of vaginal infection than BV-negative women (33.5% vs. 27.9%), over two-thirds of women in the cohort remained asymptomatic. This predominance of asymptomatic infection reinforces the recognised occult nature of BV and is consistent with global evidence showing that most BV cases occur in the absence of overt clinical symptoms. Similar findings were observed in some studies in Ghana, Ethiopia and Kenya where BV was common among asymptomatic women [6,28,29]. These findings collectively underscore the inadequacy of symptom-based case detection in this setting and support the need for routine microbiological BV screening as part of antenatal care.

Nugent scoring characterizes bacterial morphotypes rather than clinical manifestations of bacterial vaginosis as defined by Amsel’s criteria. Consequently, Nugent-defined BV may include women with clinically asymptomatic vaginal dysbiosis as well as those with clinically apparent BV. Emerging evidence further suggests that asymptomatic Nugent-defined BV may represent either a transient shift from a Lactobacillus-dominated microbiota or a relatively stable CST IV microbial community [30,31]. Because our study relied on a single Gram stain assessment without molecular microbiome characterization, these biological states could not be distinguished. This limitation may partly explain why Nugent-defined BV was not significantly associated with adverse birth outcomes in this cohort. Contrary to our hypothesis and substantial evidence from other settings, we did not observe significant associations between BV and adverse pregnancy outcomes in our cohort. The rates of preterm birth (9.0%), low birthweight (12.5%), and foetal loss (3.3%) did not differ significantly between BV-positive and BV-negative women. These findings diverge from meta-analyses and systematic reviews that have consistently demonstrated associations between BV and preterm birth [15,32–34].

Several explanations may account for these differences. First, BV diagnosed using Nugent scoring captures morphotypes but not specific pathogenic subgroups. Recent genomic studies demonstrate that only certain BV-associated organisms particularly *Gardnerella* clade 2, *Fannyhessea vaginae*, and high-density sialidase-producing strains are strongly linked to prematurity [35]. Conventional Nugent scoring therefore may be insufficient to identify high-risk infections. Secondly, pregnancy outcomes in Africa are influenced by a wide range of competing morbidities such as malaria, anaemia, hypertensive disorders, and socioeconomic hardship [14]. These conditions may overshadow or dilute the observable effect of BV on birth outcomes. Lastly, the timing of BV assessment may be critical. Although this study captured BV before 20 weeks gestation, repeated sampling throughout pregnancy may provide better insight into microbial instability, as shown in recent multi-timepoint microbiome studies.

Despite the lack of association with adverse outcomes in our study, the high prevalence of BV (30.1%) in early pregnancy has important implications for antenatal care in Ghana. Yet, despite the consistently high BV burden, evidence linking BV and prematurity in the region remains inconclusive. Our findings contribute important local evidence showing that BV alone may not be a dominant driver of birth outcome in the middle belt of Ghana. This suggests the need for integrated antenatal strategies that address the broader landscape of maternal infections and non-infectious risk factors.

However, the strong sociodemographic associations identified in our study can inform targeted education and counseling. Healthcare providers should be aware that younger women, unmarried women, and those with certain sexual behavior patterns are at elevated risk for BV. Counseling on risk reduction strategies, including limiting new sexual partners and avoiding disruption of vaginal microbiota, should be incorporated into antenatal care, particularly for high-risk groups.

### Strengths and limitations

Key strengths of this study include its large sample size, standardized Nugent-based BV diagnosis, and integration within a prospective pregnancy cohort with systematic follow-up. Limitations include the use of a single time-point BV measurement, inability to characterize BV subtypes, and potential unmeasured confounders such as micronutrient deficiencies or co-existing reproductive tract infections.

## Conclusion

BV remains highly prevalent among pregnant women in Ghana. While younger age and unmarried status were key predictors of BV, BV detected early in pregnancy was not significantly associated with preterm birth, low birthweight, or foetal loss. Future research should incorporate molecular microbiome techniques to identify high-risk BV subtypes and evaluate their contribution to adverse pregnancy outcomes in African populations.
